# Results; and Proposed Chair of Tuberculosis

**Published:** 1919-08-30

**Authors:** 


### WELSH NATIONAL MEMORIAL.

### Results; and Proposed Chair of Tuberculosis.

We alluded in our issue of August 9 to the invitation which has been made to the King by the Welsh National Memorial Association to open next spring the two sanatoria for North and South Wales at Llangwyfan and Pontywal. With their completion it is interesting to review the work accomplished by the Association hitherto, and especially since 1914. Statistics give a convenient summary. Since July 1914 the hospital beds belonging to the Association have increased from 87 to 473, sanatorium beds from 148 to 446. In the same period 11,402 patients have been treated in hospitals, 6,373 in sanatoria, and over 50,000 patients have been examined at dispensaries and visiting stations.

To give the above figures meaning, the results must be compared with the work here recorded. Mr. D. W. Evans, the General Director, has drafted a table to show the value of treatment in sanatoria. In June 1919 there were at work 43.5 of the patients discharged from sanatoria in June 1913; 48.7 of those discharged in June 1914, 51.1 of those discharged in June 1915, and a similar percentage of those discharged in June 1916. The percentage now at work of those discharged six years ago is lower presumably, because of relapse or death which has occurred in the interval. But to find still at work half those discharged is encouraging. The immediate problem is to maintain 1,000 beds, in spite of the fact that the cost has doubled. The president, Major David Davies, declares that the local authorities must increase their contributions if the number of beds is not to be reduced. The Association's work has been maintained by the levy of a rate for the first three years of ½d. in the £, and for the past two years of ¾d. in the £.

The Association's scheme to prevent tuberculosis includes, of course, a programme of research. Within the past two years the Medical School of Wales has included a chair of Preventive Medicine, and it is now suggested that a chair of tuberculosis should be established, and that its holder should become the Association's chief medical officer. Were this professorship established it is argued that the future general practitioners of Wales during their passage through the Medical School, would have at their disposal all the knowledge which the Association has gathered.

